# Diana Jean Kinloch Beck (1902–1956)

**DOI:** 10.1007/s00415-021-10408-w

**Published:** 2021-02-08

**Authors:** Benjamin W. Hunt, Stephen Pow, Frank W. Stahnisch

**Affiliations:** 1 Department of Psychology, University of Liverpool, Eleanor Rathbone Building, Bedford Street South, Liverpool, L69 7ZA United Kingdom; 2 Doctoral School of History, Central European University, Nador 9 ut., Budapest, Hungary; 3 Departments of Community Health Sciences and History, Hotchkiss Brain Institute and O’Brien Institute for Public Health, University of Calgary, 3280 Hospital Drive N.W., Calgary, AB T2N 4Z6 Canada

The history of surgery is often being viewed as a steady progression marked by major breakthroughs (the introduction of anaesthesia, carbolic acid, electrocauterization, etc.), which are recognized as triumphs over longstanding impediments; they eventually resulted in improved operating conditions for patients and physicians. However, the history of neurological surgery also reveals impediments of a sociocultural nature being surmounted in the course of time. Historically, women were grossly underrepresented in the medical field—particularly in brain surgery—with female participation in the formal study and practice of medicine having once been negligible. In the first part of the twentieth century, this barrier was steadily overcome although neurosurgery was indeed one of the last medical disciplines in which female participation remained very circumscribed. Even a decade ago, fewer than 10% of practicing neurosurgeons in the USA and UK were women [5]. In the case of the career of Diana Beck, we encounter an individual who made significant contributions both to neurosurgery and the overall cause of women in medicine, rather than simply being content at being permitted to practice.

**Fig. 1:**
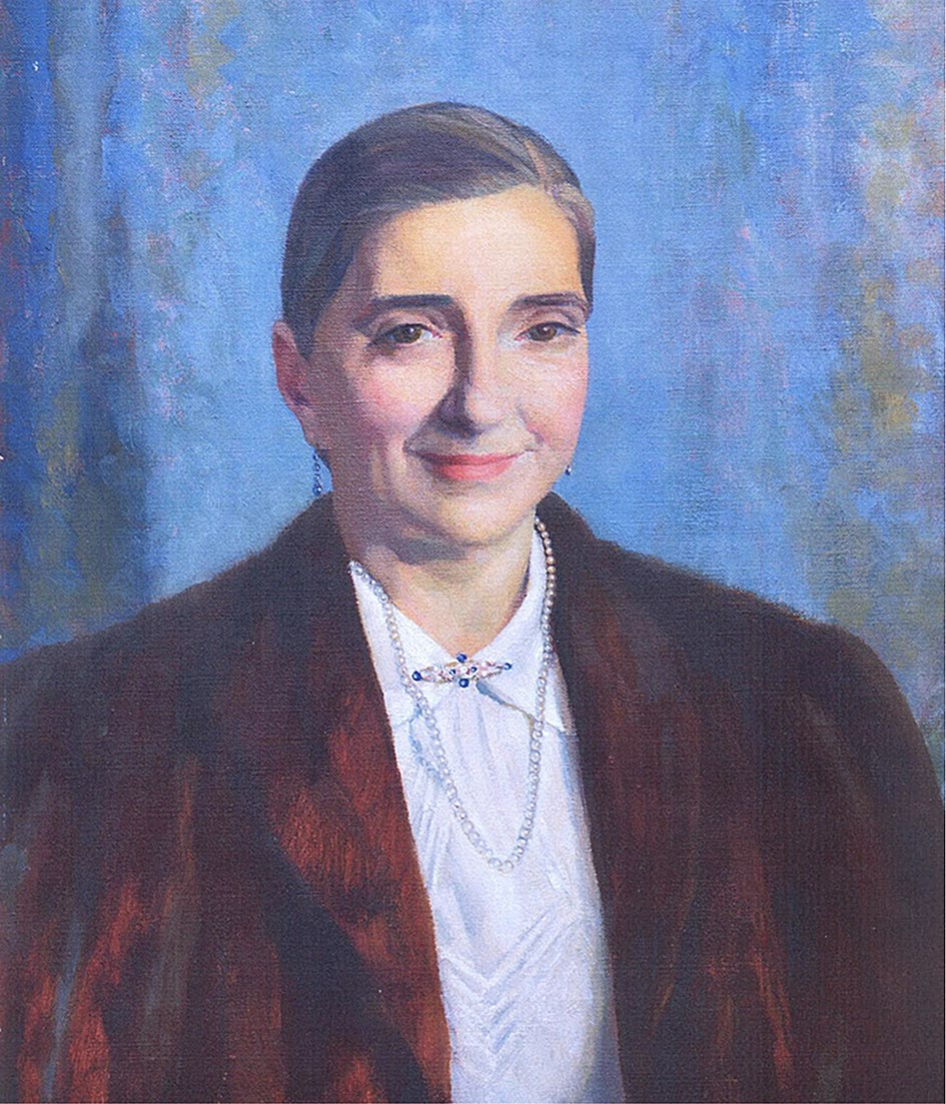
Painting of Diana Jean Kinloch Beck (1902–1956)—half-length, facing forward—produced by Phyllis Bliss (Dodd) alumna at The Queen’s School, Chester, UK

Diana Jean Kinloch Beck (1902–1956) was born on 29 June 1902, in Chester, UK, the only daughter among the three children of James Beck, a master draper, and Margaret Helena Kinloch. Her parents were of Scottish descent. She was initially educated at the reputable Queen’s School in Chester, before subsequently choosing to pursue medicine at the London School of Medicine for Women, based at the Royal Free Hospital. Beck’s university education was a distinguished one, graduating M.B., B.S. in 1925, while also earning the Julia Cock scholarship, the Gwendoline Lynn prize, and the Grant medal in surgery. Her trajectory was very unusual since at the time of Diana Beck’s birth there were fewer than 500 female doctors in both England and Wales. It was not until 1875 that several British universities were permitted to grant medical licenses to female students, thereby enabling a small number of women to graduate and ultimately participate in clinical practice [6] (Fig. 1).

Taking advantage of the relatively new historical opportunities, Beck progressed to the post of house-surgeon in the same institution where she had been educated, holding the post for 5 years. The Royal Free Hospital had been one of the earliest medical institutions to accept female students into its wards for clinical training (1877), and it would later become one of the chief neurosurgical centres in the UK during the last decades of the twentieth century [9]. From there, Beck undertook her qualification for the Fellowship of the Royal College of Surgeons, first in Scotland in 1930, and then England in the following year. In 1932, she returned to the Royal Free Hospital in London as surgical registrar and also as a lecturer in anatomy. She remained there for 7 years, becoming a popular and enthusiastic undergraduate teacher. Her Saturday rounds were seen as a highlight of the students’ experience, and students choosing to remain on the ward during weekends was not uncommon [7].

The immediate pre-war and wartime years were crucial in Beck’s neurosurgical expertise though she had already demonstrated a high ability as a general surgeon and instructor. She began a brain surgery apprenticeship in 1939 at the Radcliffe Infirmary of the Nuffield Department of Surgery in Oxford, working under prominent neurosurgeon Hugh Cairns (1896–1952) who had trained with Harvey Cushing (1869–1939) in the US and is widely considered the founding figure of British neurosurgery. From Cairns, Diana Beck inherited the “Halsted Cushing Technique”—rigorous aseptic practices prescribed by Cushing and his surgical mentor, William S. Halsted (1852–1922), which reduced sepsis and controlled bleeding. In the period before antibiotics, this technique succeeded in reducing the neurosurgical mortality rate from roughly 50% at the start of Cushing’s career to less than 10% of the patients under his care in the early 1930s [3]. As such, she was continuing in the highest practical tradition of the field, closely devoted to her mentor, and as a sign of her accomplishments, she was soon granted the William Gibson Research Scholarship, awarded by the Royal Society of Medicine in Britain. It was during this time that she collaborated with Australian pathologist Dorothy Stuart Russell (1895–1983), co-authoring, amongst others, an important work on the spread and treatment of oligodendrogliomas. Their case studies supported that these brain tumours were malignant rather than benign as had been believed previously:

“Four cases are reported in which an oligodendroglioma formed metastases throughout the cerebrospinal pathway. Two of the cases were in adults […]. The other two cases were in children: in these the clinical course was rapid and the macroscopic appearances of the metastases suggested a highly malignant glioma such as medulloblastoma.” [2]

Her first appointment as a neurosurgeon, in 1943, saw her return to the Royal Free Hospital for the third and final time. In the immediate postwar period, she became a key figure in establishing and running the neurosurgical unit at Frenchay Hospital in Bristol to treat the many sufferers of serious brain injuries from World War II. In 1947, she became the first woman to be appointed consultant neurosurgeon at the Middlesex Hospital, London, which at the time admitted only male applicants. Perhaps it can be considered a *drôle d’histoire* that she found herself teaching male students in a program to which she herself would have been unable to gain admittance. At the pinnacle of her career, she ran the hospital’s neurosurgical service. While occupying this role, she performed a lifesaving neurosurgical procedure on acclaimed children’s book author, Alan Alexander Milne (1882–1956), following a stroke in 1952—an accomplishment which attracted media attention and made her a medical celebrity in Britain [5]. Having much experience with the prognosis and recovery of patients, she published an important work while at Middlesex on the sequelae of brain injuries with the aim of improving chances of rehabilitation [1].

During the latter part of her career, in addition to her clinical practice, she also took an active academic role, publishing her research in journals and accepting presentation invitations throughout Europe, Canada, and the United States on the neurosurgical treatment of intracerebral haematomas. Based on her own clinical experience with these conditions and successful outcomes, her innovative publications on the matter offered a series of recommendations, often emphasizing early surgical treatment [10].

Diana Beck died prematurely in 1956, following a thymectomy from which a pulmonary embolism developed. Regarding her legacy, an intriguing aspect of the biography of Diana Beck is whether she ought to be considered the world’s first female neurosurgeon. In 2005, the World Federation of Neurosurgical Sciences acknowledged Sofia Ionescu (1920–2008), a Romanian physician who practiced emergency surgery during World War II, as the world’s first female brain surgeon. Ionescu’s first documented emergency neurosurgical operation took place in 1944, apparently 8 years before Beck was to perform her own first intracranial procedure [4]. However, those in favour of considering Beck as the “first,” note that Ionescu officially graduated from medical school in 1945. Whether one can be considered a practicing neurosurgeon without academic qualification credentials is a difficult question. Moreover, Beck was already a certified neurosurgeon in 1943. Regardless, one might caution against discussing these complex developments with superlatives and “firsts.” A thoughtful report in her obituary in the *Lancet* described Beck’s unique accomplishment as her having been “one of the first women to be appointed to the consultant staff of a teaching hospital that was not her own” [8]. Debates aside, one can state that Diana Beck in 1939 was almost certainly the first female to have formally trained as a neurological surgeon, and the first to have performed neurosurgery in both Western Europe and North America [11]; such accomplishments require no exaggeration.
